# Self-perceived Difficulties With Suicidal Patients in A Sample of Italian General Practitioners

**DOI:** 10.4021/jocmr684w

**Published:** 2011-11-10

**Authors:** Stefano Zanone Poma, Antonello Grossi, Emanuele Toniolo, Vincenzo Baldo, Diego De Leo

**Affiliations:** a Department of Mental Health-Local Health Authority (ULSS 18) of Rovigo, Italy; b Department of Environmental Medicine and Public Health, Institute of Hygiene, University of Padua, Italy; c Australian Institute for Suicide Research and Prevention, WHO Collaborating Centre for Research and Training in Suicide Prevention, Griffith University, Australia

## Abstract

**Background:**

Suicidal behaviours are relatively common among primary care patients, but suicide ideation seems to be poorly detected by GPs. The purpose of the present study is to investigate the frequency of issues related to suicidal behaviour in GPs’ setting and to inquire the level of difficulties perceived by physicians when dealing with suicidal patients.

**Methods:**

A survey on 88 GPs in Rovigo (Italy) has been conducted through the use of a self-administered questionnaire inquiring about suicidal behaviour in patients, personal history and outside professional lives.

**Results:**

Four out of 5 doctors have encountered at least a case of suicide in their professional career, and 3 out of 4 recorded at least a case of suicide attempt in a working year. The frequency of personal history of suicidal ideation/behaviour was 2.3%. One third of GPs have come into contact with suicides or suicide attempts outside the professional setting. Sixty one per cent of doctors admitted difficulties in exploring suicidal ideation, but tended to ascribe it to a reluctant attitude of patients.

**Conclusions:**

The study underscores GPs’ need of being helped in the difficult task of recognising suicidal patients.

**Keywords:**

General practitioner; Suicide ideation; Suicide; Suicide attempt

## Introduction

Stemming from the study conducted in the Swedish island of Gotland [1], it has become evident that general practitioners may play a first-line role in the prevention of suicidal behaviours [2]. People who kill themselves are estimated to have had a contact with a general practitioner in 50% and 80% of cases if considering, respectively, the month and the year before the suicide [3]. The frequency of these ”last contacts“ appears to be higher in elderly people [4].

Suicidal ideation and suicidal behaviours are relatively frequent in general population, even if with considerable variations in prevalence data depending on the site of investigation [5-7]. A study conducted in a sample of primary care patients has registered the presence of suicidal thoughts in 2.44% of patients [8].

Even if suicidal behaviours may result quite common in patients attending primary care facilities, GPs seem to have difficulties in recognise and manage them [9,10]. Some physicians might avoid ”suicide“ matters for the fear of worsening any possible suicidal feelings in the patient; others could feel not confident enough in their personal skills to treat this kind of subjects [11]. Some of these difficulties can be partially attributed to the relatively low importance given to courses on suicide prevention within medical school curricula [12].

Many researches have proven the efficacy of training and education programmes for GPs focused on the appropriate recognition and treatment of depressive symptoms [13,14]. In particular, it seems that education programmes addressed to patients with some psychological distress are more incisive than those based on general screening procedures [2,15]. An interesting study by Feldman et al [16] has investigated the depth of exploration of suicidal ideation by GPs on patients referred for depressive symptoms. The authors found that suicidal ideation was clearly sought only by 36% of GPs. Explicit questions on suicide were asked more frequently to patients with symptoms consistent with a diagnosis of major depression than in those with a diagnosis of adjustment disorder or in case patients clearly requested a pharmacological treatment with antidepressants. The same study also pointed out that physicians with direct experience of depression (whether at personal level or affecting a family member or a friend) were more prone to explore suicidal ideation [16]. In a following investigation by the same group of researchers [17], by audio-recording GPs’ visits, authors found that most inquiries on suicidal ideation were sensitive, appropriate and supportive.

Aim of the present study is to investigate the frequency of issues related to suicidal behaviour in GPs’ setting and to inquire the level of difficulties perceived by physicians when dealing with suicidal patients. The survey has been conducted on GPs in Rovigo, a province of northern Italy with a quite uniform and stable population (a rural area with prevailing agriculture and small industrial activities, and a relatively low rate of immigration).

## Materials and Methods

In occasion of a two-day course on the topic of suicide prevention, a survey was conducted on the general practitioners who were in attendance. Eighty-eight physicians, out of a total of 139 GPs operating in the province of Rovigo (63.3%), took part to the project. All 88 participants filled-in a self-administered questionnaire (in an anonymous way) containing three main sections:

1) A group of questions about professional life (number of patients under their care; number of years in the job) and suicidal behaviour in their patients (number of suicide cases during professional life; number of suicide attempts witnessed in a working year; perceived difficulties in exploring suicidal ideation and related reasons; educational needs on this subject).

2) Questions about personal life (gender, age, area of birth, mother-tongue) and direct experience of suicide and/or suicide attempt(s) among family members, friends or colleagues and emotional impact in relation to it; and questions about personal experience of suicide plans and/or suicide attempt(s) in own lives.

3) Third section: this part was to be filled only by those physicians declaring personal history of suicidal behaviour (method chosen, triggering events, help-seeking behaviour, etc.).

Statistical analysis was performed using SPSS software, version 16. Chi-square test (Mantel Haenszel), Fisher’s exact probability test, and Student’s t-test were chosen when appropriate. A p value of < 0.05 was considered as significant, and Odds Ratios (OR) with a 95% Confidence Interval (CI) was calculated for each parameter. A multivariate logistic regression analysis was carried out to determine variables independently associated with the variable ”difficulties in exploring suicidal ideation“.

## Results

As said, the sample was composed by 88 physicians, whose mean age was 53.8 years (SD ± 7.2), with 51 males and 37 females (average age of 55.3 and 51.6 years, respectively). Only three people were of non-Italian mother-tongue. Most physicians were born in their present working area (59.1%) or in other areas of northern Italy (33.0%). Average years of primary care practice were 22.6 (± 8.3); on average, each doctor followed 1238 patients (± 352.8); eighty-four percent of the GPs had been always working in the same territory.

Outside professional settings, 32 physicians (36.4% of the sample) have known people who died for suicide among family members, friends or colleagues. They rated the emotional impact of these events as ”high“ in 74.1% of cases, ”moderate“/“mild“ in 22.2% of cases, and ”no impact“ in only 3.7% of cases. In regard to suicide attempts, 28 physicians (31.8% of the sample) have known people who have tried to take their own lives at least once. They rated the impact of these events as ”high“ in 50.0% of cases, ”moderate“/“mild“ in 38.5% of cases, and ”no impact“ in 11.5% of cases. On average, these experiences occurred approximately 15 years before the survey, when GPs had a mean age of 40.9 and 37.4 years (for suicide and suicide attempts, respectively).

Only 62 of the 88 physicians answered the question about suicide cases among their patients; 49 of them (about 4/5) recorded at least one suicide case, with some doctors reporting up to 10 cases. Considering the question about suicide attempts in a working year, 70 GPs answered to it: 3 out of 4 usually see at least one case per year (Table 1).

**Table 1:** Frequency of Cases of Suicides and Suicide Attempts Indicated by GPs During, Respectively, Their Professional Career or A Working Year

**Seen during the professional career**		****	**In a working year**	
		
**Number of** **suicide**	**Number of physicians** **and percentage**		**Number of** **suicide attempts**	**Number of physicians** **and percentage**
None	13 (20.7%)		None	18 (25.7%)
1	24 (38.7%)		1	33 (47.2%)
2	9 (14.5%)		2	17 (24.3%)
3	8 (12.9%)		7	1 (1.4%)
4	2 (3.3%)		10	1 (1.4%)
5	2 (3.3%)		Total	70
6	2 (3.3%)			
10	2 (3.3%)			
Total	62			

**Figure 1:**
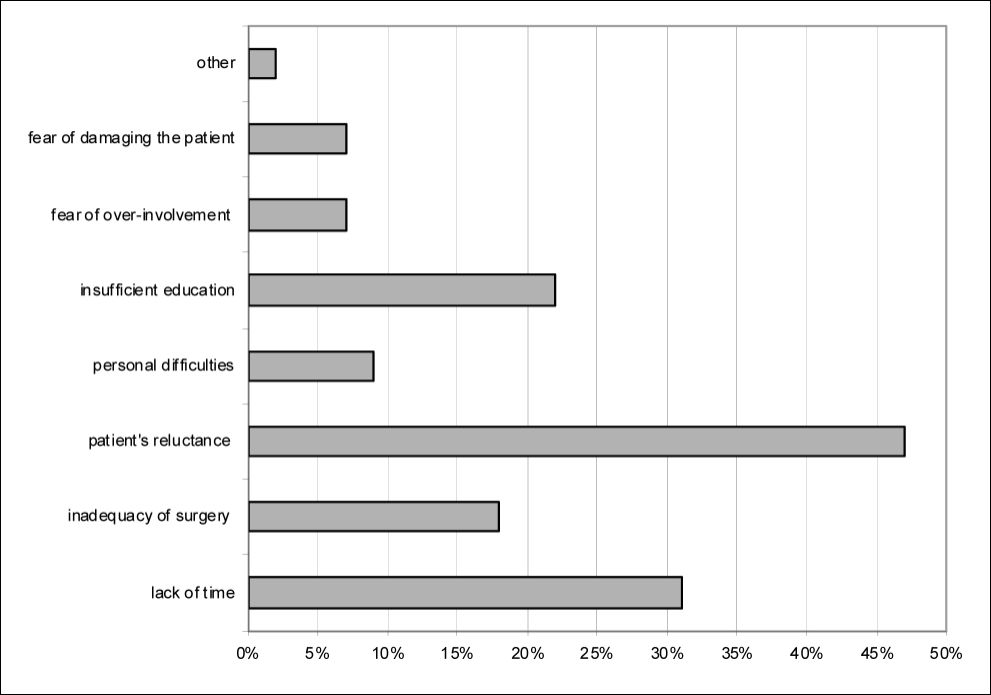
Frequency in percentage of reasons of difficulties to explore suicidal ideation (more than an option could be indicated).

Sixty-one percent of the total sample (n = 88) reported difficulties in exploring suicidal ideation in patients. The main reason for this was identified in patient’s reluctance to disclose own feelings (46% of doctors). In decreasing order of importance, other causes were lack of time, insufficient education, and inadequacy of surgery; in smaller percentages (below 10%), some doctors indicated personal difficulties, fear of over-involvement or acting inappropriately, damaging the patient (Fig. 1).

**Figure 2:**
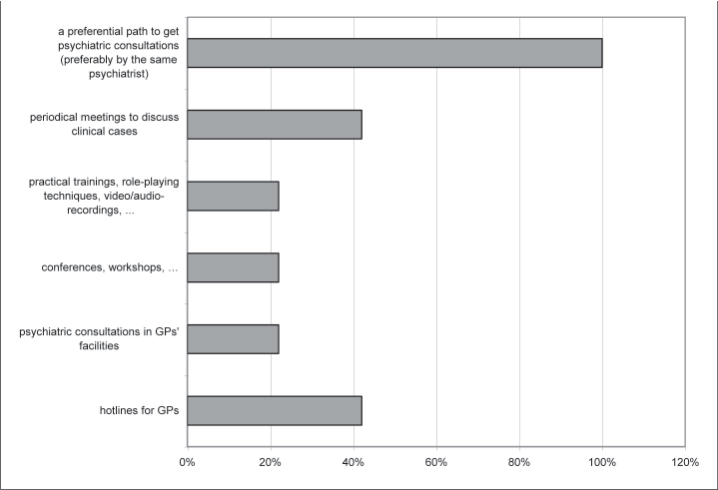
Frequency in percentage of offsetting strategies suggested by GPs (more than an option could be indicated).

The vast majority of the interviewed sample (86.4%) considers of primary importance to get better education on managing suicidal behaviours. Also, most doctors would like to obtain psychiatric consultation on cases (preferably by the same psychiatrist). Other indications from GPs include periodical meetings to discuss clinical cases and the creation of a hotline for GPs (Fig. 2).

Only two doctors reported having planned or attempted suicide in their lives: this corresponds to a rate of 2.3% life span prevalence. Due to small numbers, analysis on the third section of the questionnaire (personal experiences of suicidal behaviour) was not performed.

Multivariate analysis between the variable ”difficulty in exploring suicidal ideation“ and other variables showed one significant correlation (Table 2): the higher the number of suicide attempts seen in a working year, the greater the difficulty in exploring suicidal ideation from GPs. On the contrary, it seems that direct experience of suicides and suicide attempts outside the professional setting might reduce the difficulties (however, this association did not reach statistical significance: P < 0.06 for both scenarios).

**Table 2:** Difficulties in Exploring Suicidal Ideation: Multivariate Analysis

**Variable**	**Adjusted OR** **(95% CI)**	**P**
Gender: female versus male	1.07 (0.19-5.93)	0.94
Cases of suicide attempt among family members, friends or colleagues (no versus yes)	5.44 (0.94-31.26)	0.06
Cases of suicide among family members, friends or colleagues (no versus yes)	4.07 (0.92-17.97)	0.06
Age	0.99 (0.78-1.25)	0.92
Years of professional career	1.04 (0.87-1.23)	0.66
Cases of suicide during the professional career	0.74 (0.51-1.06)	0.10
Cases of suicide attempt in a working year	4.26 (1.48-12.28)	0.01

## Discussion

The sample investigated was rather uniform in terms cultural and geographical background and in professional characteristics. The frequency of personal history of suicidal ideation/behaviour found in the sample of physicians was 2.3%, a rate in line with the one observed in the Italian general population sample of the study ESEMED [5], but lower than the rate detected in other geo-cultural contexts [7]. With some variability, most studies indicate that medical professionals are at greater risk for suicide than the general population, and this seems particularly true in women [18]. To date, no data on prevalence of suicidal behaviour in the general population of the specific area under study are available; so, no comments can be addressed in this direction. On the other hand, participating physicians could have had some hesitations in answering to personal questions, as possibly suggested by the low number of responses related to professional experiences of suicide and attempted suicide (62 and 70 out of 88, respectively).

Essentially, suicidal behaviour appears to be a rather common phenomenon in clinical practice, given the fact that four out of five doctors have encountered at least one case of suicide in their professional career and three out of four recorded at least one case of suicide attempt in a working year. Suicidal events are also relatively common outside professional settings: about one third of the interviewed GPs have come into contact with suicides or suicide attempts among family members, friends or colleagues. These events were of high emotional impact for more than 50% of doctors (for suicides, almost 80%). Combining the data about professional and non-professional experiences, in the whole sample only five doctors resulted ‘untouched’ by suicidal behaviour. If we consider memory recall bias and possible presence of unreported data, it seems plausible to hypothesize that every GP have had some experience of this phenomenon in his/her live. This appears in line with literature [12].

However, despite the frequency of suicidal behaviour in clinical practice [3,19], most physicians find difficulties in coping with it. In our sample, 61% of doctors admitted that exploring suicidal ideation is problematic, but tended to ascribe the phenomenon to patients, in particular to their reluctance to disclose inner feelings. In addition, only few doctors declare personal difficulties or fear of over-involvement, while technical/contingent reasons are emphasised (lack of adequate qualification, time and space).

It is somehow surprising the correlation found between professional experience of suicide attempts and difficulty in exploring suicidal ideation: it could be expected that the frequency of a given event generate confidence and experience with its management. As a possible explanation, one might consider that the psychological impact of such event perturbs both self-confidence and professional management of the doctor, but it is also likely that the relatively low frequency of the events reported (on average: 1.2 in a year) does not allow for strong interpretations. Contrarily, direct experience of suicidal behaviour in non-professional settings, probably due to their (greater) emotional impact, might increase the willingness in doctors to inquire about suicide ideation. However, whilst concordant with previous research [16], this observation did not reach statistical significance.

Overall, the sample of physicians indicated the need for better education on the topic of suicide prevention, but they also expressed the desire for suicidal behaviour to be managed by specialists (e.g., a preferential path to get psychiatric consultation, hotlines for GPs, etc.). This seems to point to the need for support in such a delicate matter, but maybe also to some reluctance in getting personally involved.

In addition to all drawbacks that are implicit in surveys based on questionnaires (memory recall bias, social desirability, etc.) [20], the value of the present survey is limited by the relatively small sample of participants and the context in which the investigation was performed: physicians filled-in the questionnaire during an educational course, and the fear of being identified (despite the guarantee of anonymity) and/or the emergence of unpleasant memories (whether professional or personal) might have biased the results. In addition, the representativeness of physicians might be questioned: despite constituting a numerically sound sample (88 out of a total of 139), the hypothesis that only the physicians (more) sensitive to the subject have attended the course cannot be dismissed.

In spite of the above limitations, the present study confirms and emphasises the need of general practitioners to be helped in the difficult task of recognising and dealing with suicidal ideation in their patients. This is particularly relevant knowing that a significant proportion of people suffering psychological distress preferentially consult their GPs rather than mental health professionals [2]. For this reason, primary-care physicians play a key role in the identification and first-line treatment of suicidal patients. Thus, giving prominence to it by strengthening GPs’ abilities seems to be worth the effort [21], especially in light of previous studies that have proven the capability of GPs to perform sensitive and appropriate interviews with suicidal patients [17]. Availability of specialist consultation/assistance seems to be equally necessary; as a matter of fact, even a simple phone call can hold suicidal ideation down and possibly lessen the risk of repetition of suicide attempts [22].
